# Dl-3-n-Butylphthalide Treatment Enhances Hemodynamics and Ameliorates Memory Deficits in Rats with Chronic Cerebral Hypoperfusion

**DOI:** 10.3389/fnagi.2017.00238

**Published:** 2017-07-26

**Authors:** Zhilin Xiong, Weibiao Lu, Lihui Zhu, Ling Zeng, Changzheng Shi, Zhen Jing, Yonghui Xiang, Wenxian Li, Chi Kwan Tsang, Yiwen Ruan, Li’an Huang

**Affiliations:** ^1^Department of Neurology, The First Affiliated Hospital, Jinan University Guangzhou, China; ^2^GHM Institute of CNS Regeneration (GHMICR), Jinan University Guangzhou, China; ^3^Department of Radiology, The First Affiliated Hospital, Jinan University Guangzhou, China; ^4^Clinical Neuroscience Institute, The First Affiliated Hospital, Jinan University Guangzhou, China; ^5^Co-innovation Center of Neuroregeneration, Nantong University Nantong, China; ^6^Ministry of Education, CNS Regeneration International Collaborative Laboratory, Jinan University Guangzhou, China; ^7^Department of Anatomy, Jinan University School of Medicine Guangzhou, China

**Keywords:** DL-3-n-butylphthalide, bilateral common carotid artery occlusion, MRI, cerebral blood flow, vertebral artery, angiogenesis, cognition impairment

## Abstract

Our previous study has revealed that chronic cerebral hypoperfusion (CCH) activates a compensatory vascular mechanism attempting to maintain an optimal cerebral blood flow (CBF). However, this compensation fails to prevent neuronal death and cognitive impairment because neurons die prior to the restoration of normal CBF. Therefore, pharmacological invention may be critical to enhance the CBF for reducing neurodegeneration and memory deficit. Dl-3-n-butylphthalide (NBP) is a compound isolated from the seeds of Chinese celery and has been proven to be able to prevent neuronal loss, reduce inflammation and ameliorate memory deficits in acute ischemic animal models and stroke patients. In the present study, we used magnetic resonance imaging (MRI) techniques, immunohistochemistry and Morris water maze (MWM) to investigate whether NBP can accelerate CBF recovery, reduce neuronal death and improve cognitive deficits in CCH rats after permanent bilateral common carotid artery occlusion (BCCAO). Rats were intravenously injected with NBP (5 mg/kg) daily for 14 days beginning the first day after BCCAO. The results showed that NBP shortened recovery time of CBF to pre-occlusion levels at 2 weeks following BCCAO, compared to 4 weeks in the vehicle group, and enhanced hemodynamic compensation through dilation of the vertebral arteries (VAs) and increase in angiogenesis. NBP treatment also markedly reduced reactive astrogliosis and cell apoptosis and protected hippocampal neurons against ischemic injury. The escape latency of CCH rats in the MWM was also reduced in response to NBP treatment. These findings demonstrate that NBP can accelerate the recovery of CBF and improve cognitive function in a rat model of CCH, suggesting that NBP is a promising therapy for CCH patients or vascular dementia.

## Introduction

Chronic cerebral hypoperfusion (CCH) occurs in the early stages of senile dementia including Alzheimer’s disease (AD), vascular dementia (VaD) and mixed dementia (Pappas et al., 1996; Yoshikawa et al., 2003; Schuff et al., 2009; Zhao and Gong, 2015), which has been considered to play an important role in neurodegeneration in the development of dementia and AD. It has been reported that many diseases such as heart disease (de la Torre, 2012), hypertension (Farkas et al., 2002; Choi et al., 2015), diabetes (Kwon et al., 2015) and atherosclerosis (McColl et al., 2009) contribute to vascular constriction and cause CCH.

The mechanisms underlying dementia caused by CCH involve neuronal loss (Pappas et al., 1996; Bang et al., 2013), white matter lesion (Tomimoto et al., 2003; Chida et al., 2011), glial activation (Simpson et al., 2007), synaptic dysfunction (Simpson et al., 2007), oxidative stress (Ritchie et al., 2004; Kašparová et al., 2005), accumulation of Aβ (Li et al., 2009b; Okamoto et al., 2012) and aggravation of progressive cognitive deficits (de la Torre, 2012; Choi et al., 2015).

Bilateral common carotid artery occlusion (BCCAO) is a widely used model of vascular dementia due to its instigation of CCH (Pappas et al., 1996; Farkas et al., 2007; Jing et al., 2015). Our previous study showed that BCCAO reduced cerebral blood flow (CBF) until 3 weeks when the CBF returned to the baseline level; however, neuronal death and cognitive impairment occurred at 2 weeks post-BCCAO in rats (Jing et al., 2015). Therefore, it is critical to recover the CBF earlier to prevent neurodegeneration and memory deficits. Unfortunately, there is currently no effective therapies available to accelerate the recovery of CBF and improve cognitive function in CCH.

L-3-n-Butylphthalide (l-NBP) was first isolated from the seeds of Chinese celery and was discovered within the last few decades. Based on the therapeutic property of this compound, dl-3-N Butylphthalide (dl-NBP, or NBP) was synthesized later in China and approved by the China Food and Drug Administration (CFDA) as an acute stage anti-ischemia treatment (Abdoulaye and Guo, 2016). NBP is a synthetic chiral compound containing L- and D-isomers of butylphthalide. Studies have demonstrated that NBP can reduce blood glucose level in diabetes (Zhang et al., 2011; Wang et al., 2016), decrease glial activation in a mouse model of amyotrophic lateral sclerosis (Feng et al., 2012), protect dopamine neurons in a rotenone model of Parkinson’s disease (Xiong et al., 2012), prevent neural loss in experimental ischemic stroke (Liu et al., 2007; Li et al., 2010), ameliorate vascular cognitive impairment in ischemic and dementia patients (Zhu et al., 2014) and AD models (Peng et al., 2010). Studies also demonstrated that NBP is safe and is extensively metabolized by multiple enzymes (Diao et al., 2013). However, its effects on CBF, glial cell reactivity and neuron degeneration following CCH remain unclear.

Based on the discovery of the effects of NBP in an acute ischemic stroke (AIS) model and in patients, we hypothesized that NBP may be also effective for CCH. In the present study, we used magnetic resonance imaging (MRI) techniques, immunochemistry and Morris water maze (MWM) to explore the effect of NBP on dynamic changes in CBF, glial cell reactivity, neurodegeneration and cognitive function in CCH rats induced by BCCAO.

## Materials and Methods

### Animals and Drug Administration

Adult male Sprague-Dawley rats (420–450 g, approximately 6 months old) were obtained from the Animal Experiment Center of Southern Medical University (Guangzhou, China). All rats were housed in cages under controlled temperature (21 ± 1°C) and humidity (55 ± 10%), with a 12 h light/12 h dark cycle. Food and water were available *ad libitum* throughout the study. All animal procedures were performed in strict accordance with the recommendations in the Guide for the Care and Use of Laboratory Animals of the National Institutes of Health. The protocol was approved by competent ethics committees at Jinan University.

Rats (94 total) were randomly divided into seven groups. Two groups: the vehicle group and the NBP-treated group, were assigned for measurement of CBF and vertebral artery (VA) diameters, and five groups (Sham, Veh- 2 weeks, NBP- 2 weeks, Veh- 4 weeks, and NBP- 4 weeks) for detection of behavior and pathology. The allocation of animals in each group is shown in Table 1.

**Table 1:** Information of animal numbers.

Animal groups	Beginning	Died during experiment	End	Experiment
Vehicle	12	4	8	CBF
NBP	12	2	10	CBF
Sham	10	0	10	Behavior
Veh- 2 weeks	15	5	10	Behavior
NBP- 2 weeks	14	4	10	Behavior
Veh- 4 weeks	16	6	10	Behavior
NBP- 4 weeks	15	5	10	Behavior

NBP-treated rats received daily tail-vein injection of NBP solution (5 mg/kg) in HP-β-CD and 9% saline for 14 days (from day 1 to 14 after BCCAO). Observation on the pathological changes in rats was made at 2 weeks (NBP- 2 weeks) and 4 weeks (NBP- 4 weeks) after BCCAO. Rats in the vehicle group received a daily tail-vein injection with the solvent (HP-β-CD and saline) in the same dose as the NBP- treated group (but without NBP) for 14 days after BCCAO. For sham group, rats were undergone the same surgical and manipulation procedures without BCCAO.

DL-3-n-butylphthalide was provided by Shijiazhuang Pharmaceutical Group Co. Ltd. NBP was dissolved in 0.9% saline with a Hydroxypropyl-β-cyclodextrin (HP-β-CD: 0.9% saline, 1:3), and prepared for intravenous injection (i.v.). The chemical structure of dL-3-n-butylphthalide 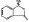 is shown as the figure on the left. NBP is a novel synthetic chiral compound containing L- and D-isomers of butylphthalide.

### Chronic Cerebral Hypoperfusion

CCH was induced by the modified permanent BCCAO (2VO) method, with initial occlusion of the right common carotid artery (RCCA) followed by occlusion of the left common carotid artery 1 week later, as described previously (Sarti et al., 2002). Briefly, rats were anesthetized with 10% chloral hydrate (0.35 mL/100 g). The animal was placed facing up on a surgery table, and a ventral midline incision was performed on the neck to expose the common carotid arteries. The RCCA was carefully separated from the vagus nerve and surrounding tissues, then ligated permanently with double sutures (3-0). During the procedure, the operating room was maintained at a temperature of 28.0 ± 2.0°C. The left common carotid artery occlusion (LCCAO) was performed in the same manner 1 week after RCCA occlusion (RCCAO). Sham-operated rats underwent the same surgery procedure except occlusion of common carotid artery.

### Magnetic Resonance Imaging

MRI experiments were conducted with a Discovery 750 3.0T scanner with an 8-channel wrist coil (GE Healthcare, Milwaukee, WI, USA). NBP-treated and vehicle group rats were scanned with MRI at six time points starting at pre-occlusion, then after BCCAO, followed by 1, 2, 3 and 4 weeks after BCCAO. Following anesthesia with 10% chloral hydrate (0.3 mL/100 g), animals were placed in a supine position prior to scanning. All imaging parameters for the 3D ASL series were described in our previous study (Jing et al., 2015). Briefly, 15 slices acquired in ascending order (slice thickness = 4 mm), no gap between slices; field of view = 120 mm × 120 mm, matrix = 512 (points) × 12 (arms); number of excitations = 5, bandwidth = 62.5 kHz, scan duration was 9 min 14 s, labeling duration = 1650 ms, post labeling delay = 1025 ms, repetition time = 4132 ms and echo time = 11 ms.

MRI images of blood vessels were captured by time-of-flight angiography with a 3D Fast SPGR. Scan parameters were as follows: echo time = 3.9 ms, repetition time = 20 ms, field of view = 80 mm × 60 mm, matrix = 320 × 224, number of excitations = 1, bandwidth = 31.2 kHz, and scan duration was 231 s.

### 3D Arterial Spin Labeling Technique

CBF in different brain regions of the rats was measured using the 3D Arterial Spin Labeling (ASL) technique. The processes and the parameters of this technique were used as described in our previous study (Jing et al., 2015).

### Morris Water Maze Task

To evaluate deficits in learning and spatial memory caused by cerebral chronic hypoperfusion, rats in each group (*n* = 10) were evaluated using a MWM at 2 weeks and 4 weeks after BCCAO using methods described by Morris (1984). Escape latency of rats to find and stand on a platform under water in navigation trials and travel path around and within the quadrant of the platform in probe trials were monitored by a video camera linked to an animal behavioral recording system (Ethovison XT, Noldus Information Technology Co., The Hague, Netherlands).

### Tissue Preparation

**Figure 1:**
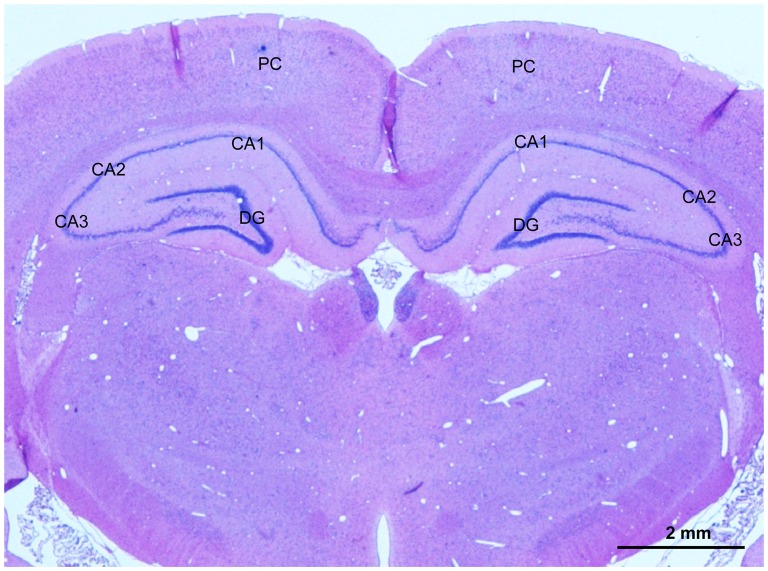
A representative photomicrography of hematoxylin and eosin (HE) stained section showing selected areas for evaluation of the angiogenesis, glial activation, apoptosis and neuronal injury in the parietal cortex (PC) and CA1 area and CA3 area in the hippocampus. Scale bar is 2 mm.

At 2 weeks and 4 weeks after BCCAO, rats (*n* = 4 in each group) were anesthetized as described above and transcardially perfused with physiologic saline followed by 4% paraformaldehyde in 0.1 mol/L phosphate-buffered saline (PBS, pH 7.4). The brain was dissected out immediately and post-fixed in the same fixative overnight at 4°C. Coronal blocks from the optic chiasm to the posterior level of hypothalamus, which includes the hippocampus and parietal cortex (PC), were prepared, and processed for dehydration with an increasing alcohol gradient and three xylene exposures in an Automated Tissue Processor (LYNX II, Hatfield, PA, USA). The blocks were embedded with paraffin in an embedding machine (Thermo Scientific HistoStar, Kalamazoo, MI, USA). Coronal sections at 10-μm-thickness were cut with a paraffin microtome (Leica, RM2235, Wetzelar, Germany). Sections were mounted on slides for staining, and one representative photomicrograph showing PC and hippocampus of these sections was taken from hematoxylin and eosin (HE) stain as shown in Figure 1.

### Immunofluorescent Labeling

Immunofluorescence was performed as previously described (Jing et al., 2015). Briefly, sections were processed for immunofluorescence labeling with CD34 antibody (for endothelial cells of microvessels), glial fibrillary acidic protein (GFAP) antibody (for astrocytes), NeuN antibody (for neurons) and cleaved caspase-3 (C-caspase 3) antibody (for apoptotic cells). First, sections were immersed in blocking solution (5% normal goat serum or donkey serum in PBS) at room temperature for 2 h, then incubated overnight at 4°C with CD34 antibody (Rabbit, 1:200, Boster, Wu Han, China), GFAP antibody (Rabbit, 1:1000, Abcam, United Kingdom) separately, and NeuN antibody and cleaved caspase-3 antibody (goat or mouse, 1:1000, Abcam, Cambridge, MA, USA) for double-labeling. After three washes with 0.01 M PBS, the sections were then incubated with secondary antibody (Alexa Fluor 488-conjugated goat anti-rabbit, donkey anti-rabbit, or donkey anti-mouse IgG and Alexa Fluor 546-conjugated donkey anti-goat, Jackson Immunoresearch, West Grove, PA, USA) for 2 h at room temperature. The concentration of all secondary antibodies was 1:1000. After three washes in PBS, sections were covered with anti-quenching fluorescence mounting medium.

### Quantitative Analysis

#### Detection of Cerebral Blood Flow

CBF was automatically calculated from 3D-ASL images by a scanner software (Functool 3D ASL, Software version 4.5, GE Medical Systems, Milwaukee, WI, USA). Approximately 2 × 2 mm regions from the PC, basal ganglion and hippocampus areas were selected for CBF measurement. A total of 48 regions from these areas in each group were analyzed at each time point.

#### Measurement of Vertebral Arteries (VAs)

Images showing VAs were captured by 3D-TOF angiography. The diameter of both VAs were measured at the middle of the cervical region. A total of 16 diameter values in each group were analyzed at each time point.

#### Analysis of Numbers of CD34, GFAP, C-Caspase 3 and NeuN Positive Cells

For pathological detection, five rats in each group were used. In each group, a total of 40 sections at 100-μm intervals at one time point were selected for immunofluorescent labeling. Digital images were captured under 400× magnifications from the CA1 and CA3 subfields of the hippocampus and PC. Forty photos were taken from both side of the CA1 region, CA3 region or PC in each group at one time point separately. The number of CD34, GFAP, C-caspase 3 and NeuN positive cells in an area (45 μm^2^) of each image was counted with ImageJ. The exposure time for imaging was consistent in each photo. Measurements were performed by two individuals blind to the study parameters.

#### Analysis of Spatial Memory Function

The data concerning escape latency and frequency of rats crossing the original platform were transferred from the animal behavioral recording system to Software Origin for analysis. A total of 40 numerical values in each group at different time points were analyzed.

### Statistical Analysis

Values presented in the study were expressed as mean ± standard error. CBF and MWM data were input to Software Origin and analyzed by repeated measures two-way ANOVA with Bonferroni *post hoc* test. The remaining non-repeated data were input into StatView software (Version 5.1) and analyzed using an unpaired-test. Differences were considered statistically significant when *p* was less than 0.05.

## Results

### NBP Promoted Recovery of CBF in the Parietal Cortex, Hippocampus and Striatum

**Figure 2:**
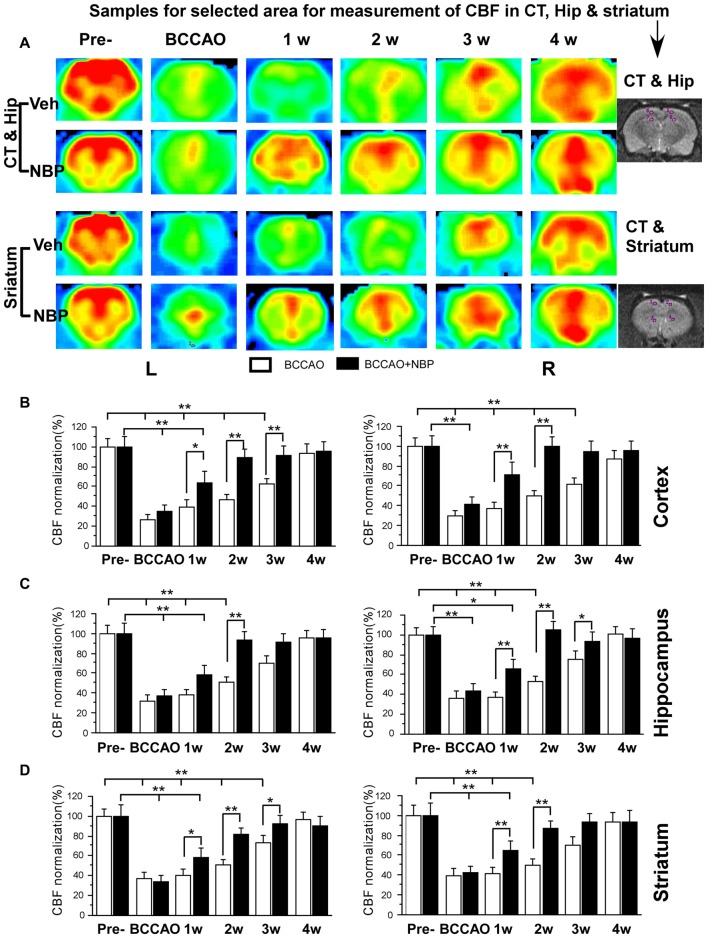
Magnetic resonance imaging (MRI; 3D ASL) analysis showing changes in cerebral blood flow (CBF) in the vehicle and NBP-treated rat brains at different time points following bilateral common carotid artery occlusion (BCCAO). **(A)** CBF of the PC and hippocampus in the vehicle group is shown in the top row and the NBP-treated group in the second row. Likewise, CBF in the striatum with vehicle treatment is displayed in the third row, and the NBP-treated group is shown in the fourth row of images. The red color labeling represents increased CBF level, while green indicates lower CBF level. CBF quantitative analysis results in the cortex **(B)** hippocampus **(C)** and striatum **(D)** in the vehicle and BNP-treated groups at different time points after BCCAO. **p < 0.01; *p < 0.05. CT, cortex; Hip, hippocampus; Pre, pre-occlusion; BCCAO, bilateral common carotid artery occlusion; L, left; R, right.

Using the 3D ASL technique, we detected CBF in the PC, hippocampus and striatum of rats in the NBP-treated and vehicle groups at the time of pre-occlusion, beginning of BCCAO, and 1, 2, 3 and 4 weeks after BCCAO. Color signals from the imaging system range from green to red and represent an increasing gradient of CBF level. Before BCCAO, red signal appeared in the brain areas including the cortex, hippocampus and striatum in both groups in the pre-occlusion condition (Figure 2A). After BCCAO, signals were shifted to the green end of the spectrum in vehicle-treated rats indicating low CBF. This persisted until 3 weeks post-occlusion when the red signal reappeared in the cortex, hippocampus and striatum and reached pre-occlusion levels at 4 weeks after BCCAO. These results demonstrated the success of this CCH model, and were consistent with our previous study (Jing et al., 2015). But in NBP-treated animals, red signal reappeared at 1 week following BCCAO and gradually became more prominent until 4 weeks after BCCAO (Figure 2A).

Quantitative analysis showed dynamic changes in CBF of different brain areas (Figures 2B–D). In the left cortex, CBF in vehicle-treated rats dramatically reduced to 27.02% ± 4.92 upon BCCAO, 39.45% ± 6.99 at 1 week, 46.68% ± 5.65 at 2 weeks and 62.37% ± 6.21 at 3 weeks after BCCAO (all *p* < 0.01 vs. the pre-occlusion level). Left cortical CBF returned to normal level at 4 weeks post-BCCAO (*p* > 0.05 vs. the pre-occlusion level; Figure 2B). However, in the NBP-treated group, CBF in the left cortex returned to 88.71% ± 8.81, 91.72% ± 8.65, 95.60% ± 9.02 from 2 weeks, 3 weeks to 4 weeks after BCCAO, respectively (all *p* > 0.05 vs. the pre-occlusion level). CBF significantly increased at these time points in the NBP-treated group compared with vehicle-treated animals (*p* < 0.05 at 1 week, *p* < 0.01 at 2 weeks and 3 weeks). In general, changes in the right cortical CBF were similar to the left cortex. In this brain region, CBF for NBP-treated animals had returned to 71.25% ± 12.68 at 1 week which was similar to the pre-occlusion level (*p* > 0.05 vs. Figure 2B). Changes in hippocampal CBF are shown in Figure 2C. In the left hippocampus, CBF was also markedly decreased to 37.26% ± 6.80 during the period of BCCAO in the vehicle group (*p* < 0.01 vs. the pre-occlusion level) and gradually recovered to 72.99% ± 8.05 at 3 weeks (*p* > 0.05 vs. the pre-occlusion level). However, after NBP treatment, CBF recovered to the normal level at 2 weeks (81.65% ± 6.46, *p* > 0.05 vs. the pre-occlusion level, and *p* < 0.01 vs. the vehicle group). The changing pattern of CBF in the right hippocampus of the vehicle group was similar with that in the left hippocampus. However, NBP treatment induced more effective outcome for the CBF, which was 64.28% ± 10.32 at 1 week and 87.45% ± 7.53 at 2 weeks (both *p* < 0.01 vs. the vehicle group). The CBF level has reached to the normal level at 2 weeks (*p* > 0.05 vs. the pre-occlusion group). For the striatum (Figure 2D), CBF changes were similar to those observed in the cortex. CBF in the left striatum dramatically reduced to 32.21 ± 5.58 during BCCAO, and gradually recovered to pre-occlusion levels (95.54% ± 7.27) at 4 weeks after BCCAO. However, after NBP treatment, the CBF level was significantly higher than that in vehicle group at 1 week (57.88% ± 9.96 vs. 38.56% ± 4.92), at 2 weeks (93.62% ± 8.18 vs. 50.77% ± 5.86) and at 3 weeks (91.7% ± 0.17 vs. 70.4% ± 6.97). The CBF of the right striatum underwent similar changes as the left striatum. Although the recovery of CBF to the normal level in the right striatum in vehicle-treated rats occurred earlier than that in the left striatum at 3 weeks after BCCAO. Therefore, the effect of NBP treatment occurred at 1 week (65.35% ± 9.65 vs. 37.27% ± 5.28) and 2 weeks (93.97% ± 8.87 vs. 53.02 ± 5.72), both *p* < 0.01 between NBP-treated rats and vehicle-treated rats.

### NBP Stimulated the Dilation of Bilateral Vertebral Arteries after BCCAO

**Figure 3:**
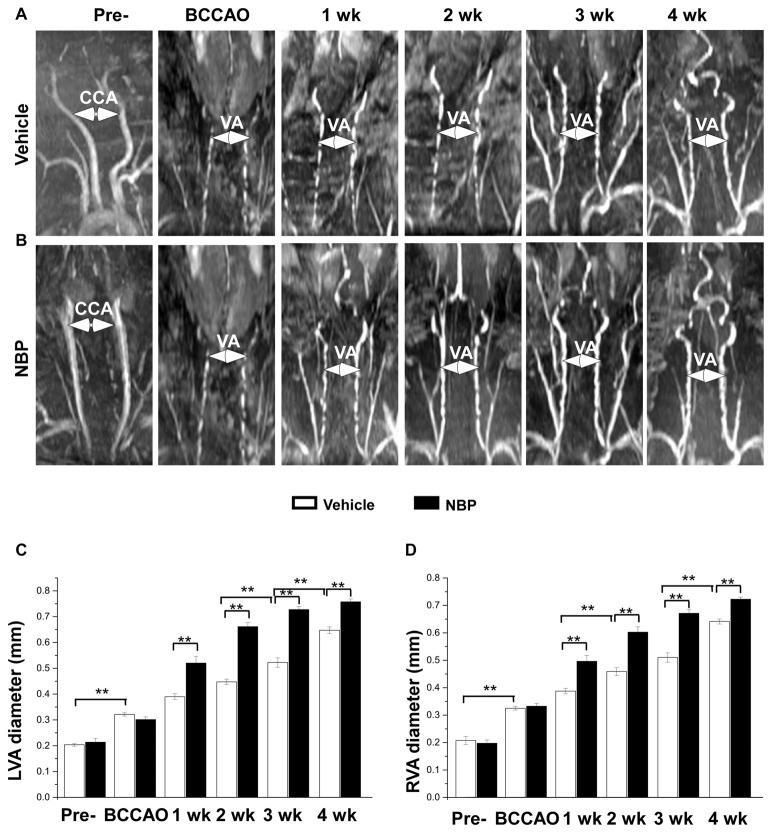
MRI (3D-TOF) angiography showing morphological changes in diameter of vertebral arteries (VAs) in the vehicle and NBP-treated groups. Morphological changes in VAs are shown in the vehicle group **(A)** and NBP-treated group **(B)**. Both CCAs were visible pre-occlusion but were not after BBCAO. However, the diameter of both VAs gradually increased until 4 weeks after BCCAO. VA diameter progressively increased after NBP treatment. **(C,D)** Quantitative analysis showing the changes of left **(C)** and right **(D)** VA diameter from 1 to 4 weeks in the NBP-treated group and the vehicle-treated group. **p < 0.01.

In our previous study, we found that the VA diameter increased after BCCAO (Jing et al., 2015). In the present study, we further investigated whether NBP would influence VAs. The results showed morphological changes of VAs in both the vehicle and NBP-treated groups at different time points after BCCAO (Figures 3A,B). Quantitative analysis indicated that the diameter of normal left VAs was 0.2 mm ± 0.01 in the vehicle-treated rats (Figure 3C), but increased to 0.32 mm ± 0.01 in response to BCCAO and gradually increased to 0.65 mm ± 0.01 at 4 weeks after BCCAO. When treated with NBP, the VAs of rats exhibited much greater dilation than that observed in the vehicle treatment group. The diameter of the left VAs of NBP-treated rats was significant larger than the vehicle group at 1 week (0.52 mm ± 0.03 vs. 0.39 mm ± 0.01), 2 weeks (0.66 mm ± 0.02 vs. 0.45 mm ± 0.01), 3 weeks (0.73 mm ± 0.01 vs. 0.52 mm ± 0.02) and at 4 weeks (0.76 mm ± 0.01 vs. 064 mm ± 0.01) post-BCCAO (all *p* < 0.01). Alteration in the value and the pattern of diameter of the right VAs in the vehicle group and NBP-treated group were similar to those observed for the left side (Figure 3D).

### NBP Induced Angiogenesis in the Cortex and Hippocampus after BCCAO

**Figure 4:**
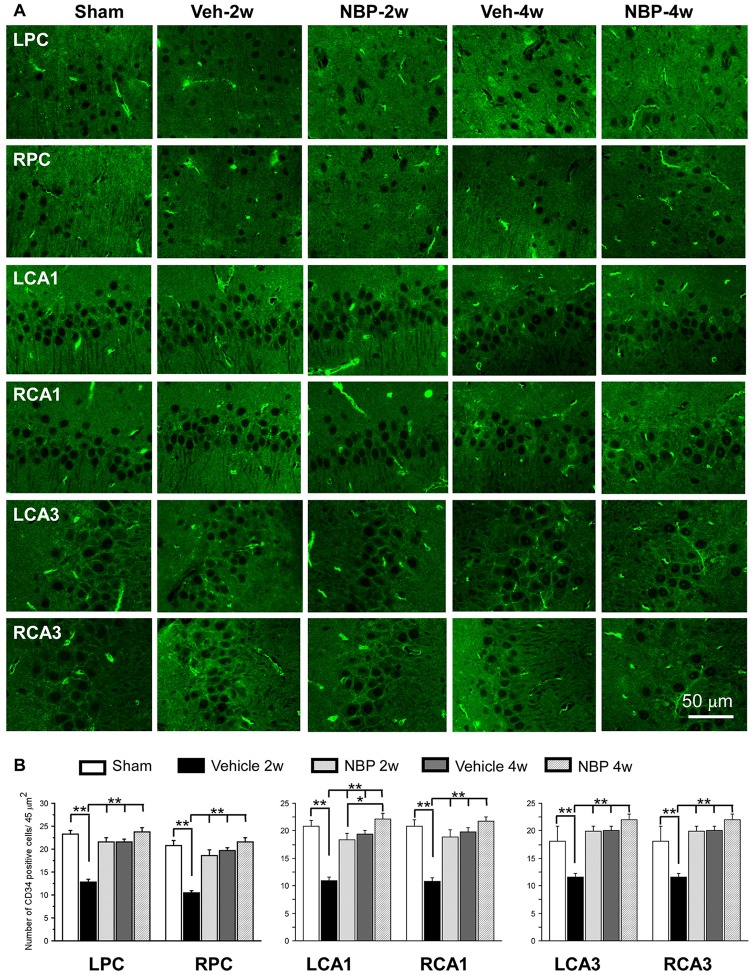
Changes in the density of immunolabeled microvessels in response to vehicle and NBP treatment. **(A)** Immunofluorescent labeling showing the density of CD34-positive microvessels in the left parietal cortex (LPC), right parietal cortex (RPC), left CA1 area (LCA1), right CA1 area (RCA1), left CA3 area (LCA3) and right CA3 area (RCA3) in the sham group, the vehicle group at 2 weeks after BCCAO (Veh-2 weeks), vehicle group at 4 weeks after BCCAO (Veh-4 weeks), BNP-treated group at 2 weeks after BCCAO (NBP-2 weeks) and NBP-treated group at 4 weeks after BCCAO (NBP-4 weeks). **(B)** Quantitative analysis showing changes in microvessel density in these areas at different time points after BCCAO. The scale bar in the image of the lower right corner is also contributed to other images in **(A)**. **p < 0.01; *p < 0.05.

To investigate the effect of NBP on angiogenesis after BCCAO, we used CD34 (an endothelial cell marker) immunofluorescence labeling to detect changes in angiogenesis in the PC, the CA1 area and the CA3 region of the hippocampus at 2 weeks and 4 weeks after BCCAO (Figure 4A). Quantitative analysis data is shown in Figure 4B. In these three areas, changes in the number of CD34 positive cells exhibited a similar trend that hypoperfusion induced a significant decrease in the number of CD34 positive cells significantly at 2 weeks (*p* < 0.01, vs. sham) but returned it to the normal level at 4 weeks (*p* > 0.05, vs. sham) after BCCAO. However, NBP treatment prevented the decrease of CD34 positive cells at 2 weeks after BCCAO (*p* > 0.05, vs. sham).

### NBP Reduced Astrocyte Activation in the Hippocampus in CCH Rats

**Figure 5:**
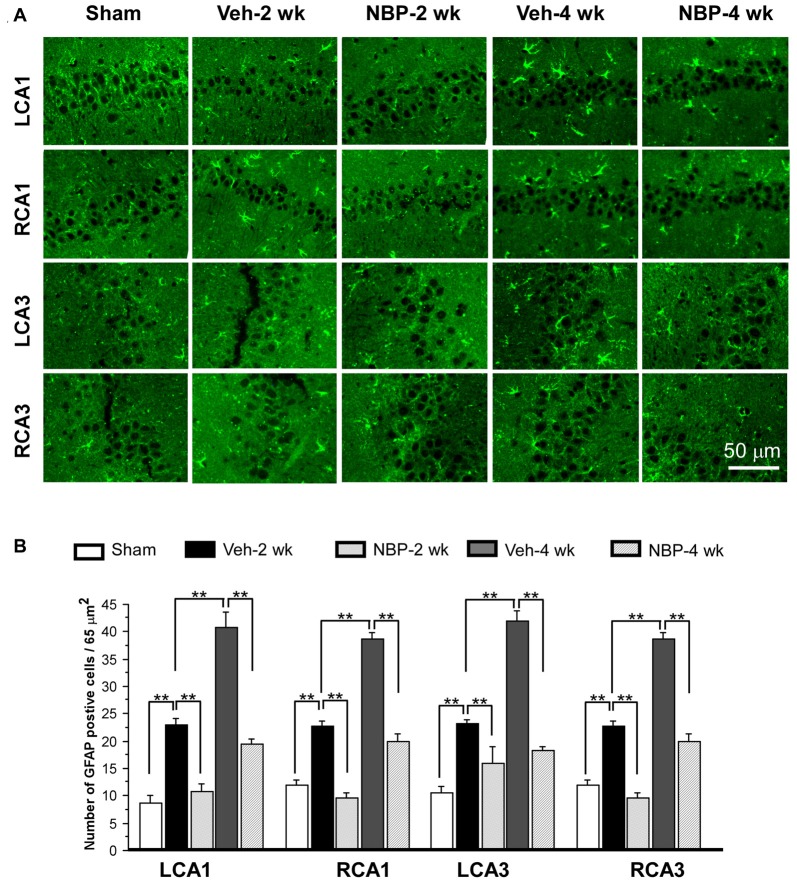
Changes in astrocyte reactivity in response to vehicle and NBP treatment. **(A)** Immunofluorescent labeling shows glial fibrillary acidic protein (GFAP)-positive cells in LCA1, RCA1, LCA3 and RCA3 in sham animals, as well as Veh-2 week, Veh-4 week, NBP-2 week and NBP-4 week groups. **(B)** Quantitative analysis indicates changes in astrocyte density in these areas at different times following BCCAO. The scale bar in the image of the lower right corner is also contributed to other images in **(A)**. **p < 0.01.

To investigate the neuroprotective effects of NBP, we employed GFAP single labeling or NeuN and cleaved-caspase-3 double labeling techniques to identify astrocytes, neurons and apoptosis cells, respectively, in the hippocampus. The temporal changes in GFAP immunofluorescent labeling in each group are shown in Figure 5A. Quantitative analysis demonstrated that the number of GFAP positive cells/45 μm^2^ was 8.75 ± 1.44 (LCA1) and 11.87 ± 1.05 (RCA1) in the sham group (Figure 5B). This number was increased to 23.00 ± 1.03 (LCA1) and 22.75 ± 0.92 (RCA1) in the vehicle group at 2 weeks, and 40.75 ± 2.87 (LCA1) and 38.75 ± 1.21 (RCA1) at 4 weeks after BCCAO (all *p* < 0.01 vs. the sham group). Following NBP treatment, the number of GFAP positive cells markedly decreased to 10.75 ± 1.42 (LCA1) and 9.50 ± 1.01 (RCA1) at 2 weeks and 19.50 ± 0.86 (LCA1) and 20.00 ± 1.41 (RCA1) at 4 weeks following BCCAO (both *p* < 0.01 vs. the vehicle group). For the CA3 area, the number of GFAP positive cells and temporal pattern of change in both sides were similar to that in the LCA1 area after BCCAO among the sham, the vehicle and NBP-treated groups (Figure 5B).

### NBP Reduced Apoptosis and Protected Neurons in the Hippocampus in CCH Rats

**Figure 6:**
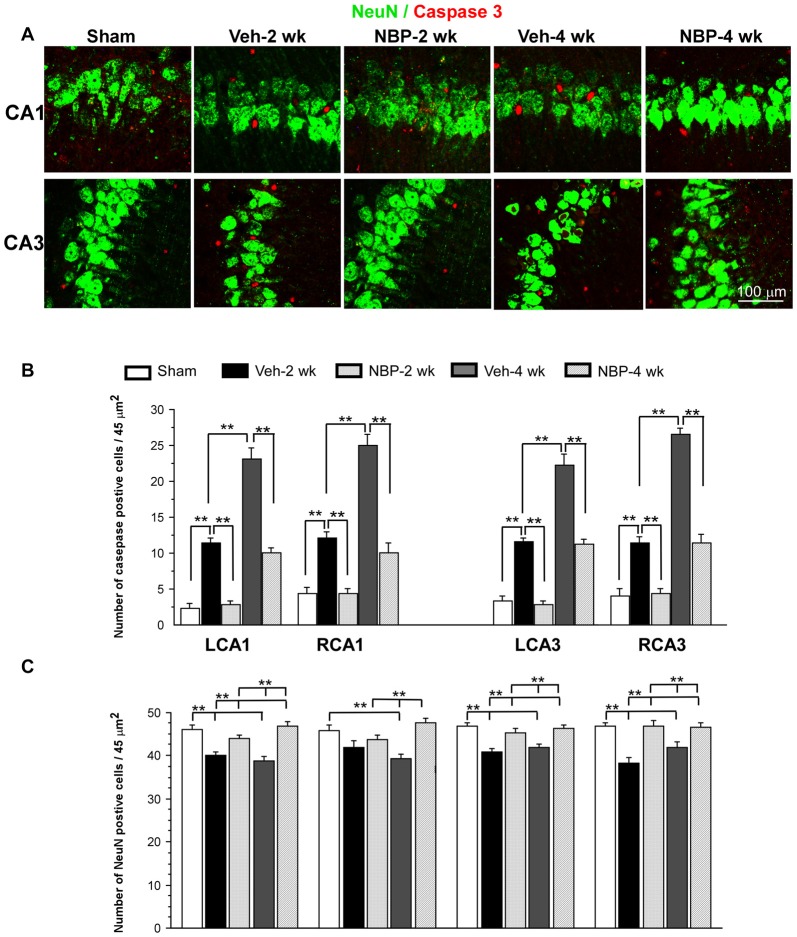
Effect of NBP treatment on neurodegeneration. **(A)** Representative images of cleaved caspase-3 and NeuN positive cells in the left CA1 and CA3 areas in the sham, Veh-2 week, Veh-4 week, NBP-2 week and NBP-4 week groups. **(B,C)** Quantification revealed changes in the number of Caspase-3 and NeuN positive cells in the LCA1, RCA1, LCA3 and RCA3 regions at different time points post-BCCAO. The scale bar in the image of the lower right corner is also contributed to other images in **(A)**. **p < 0.01.

Representative images of double labeled neuronal (NeuN positive, green) and apoptotic markers (cleaved-Caspase-3 positive, red) in the left CA1 and CA3 area are shown in Figure 6A. Quantitative analysis indicated that the number of caspase-3 positive cells/45 μm^2^ markedly decreased to 2.5 ± 0.50 (LCA1) and 3.87 ± 0.71 (RCA1) at 2 weeks after BCCAO in the NBP-treated group, which was near sham level in the LCA1 (2.00 ± 0.65) and RCA1 (3.87 ± 0.82) but significantly less than that observed in the vehicle group in the LCA1 (10.25 ± 0.67, *p* < 0.01) and the RCA1 (11.00 ± 0.71, *p* < 0.01, Figure 6B). Although the number of caspase-3 positive cells in the LCA1 (9.12 ± 0.61) and RCA1 (9.00 ± 1.28) was still greater than the sham level (both *p* < 0.01) at 4 weeks after BCCAO in the NBP-treated group, it was significantly reduced compared to the numbers in the LCA1 (21.00 ± 3.85) and RCA1 (22.62 ± 1.28) in the vehicle-treated group at this time point (both *p* < 0.01). Changes in number and pattern of caspase-3 positive cells over time in both sides of the CA3 area were similar to the LCA1 area in both the vehicle and NBP-treated groups at different time points post-BCCAO (Figure 6B).

Change in the number of neurons (NeuN positive cells) in the CA1 and CA3 regions was shown in Figure 6C. The number of NeuN positive cells in the left CA1 area in vehicle-treated rats significantly decreased when compared to sham rats (*p* < 0.01) but significantly increased after NBP treatment when compared to the vehicle-treated rats (*p* < 0.01) at 2 weeks and at 4 weeks after BCCAO. In addition, there was no difference between the NBP-treated rats and the sham rats (*p* > 0.05) at these postischemic time points. In the right CA1 area, the number of NeuN positive cells markedly decreased at 4 weeks after BCCAO but it was significantly recovered to the normal level in NBP-treated rats at the same postischemic time point. Also, there were no difference in the number of NeuN positive cells between the sham rats and BNP-treated rats at 2 weeks or 4 weeks following BCCAO. Both left and right CA3 areas shared a similar changing pattern that the number of NeuN positive cells significantly decreased in vehicle-treated rats (*p* < 0.01 vs. the sham rats) but returned to the sham level in NBP-treated rats (*p* < 0.01 vs. the vehicle rats) at 2 weeks and 4 weeks following BCCAO.

### NBP Ameliorated Learning and Memory Deficits after BCCAO

**Figure 7:**
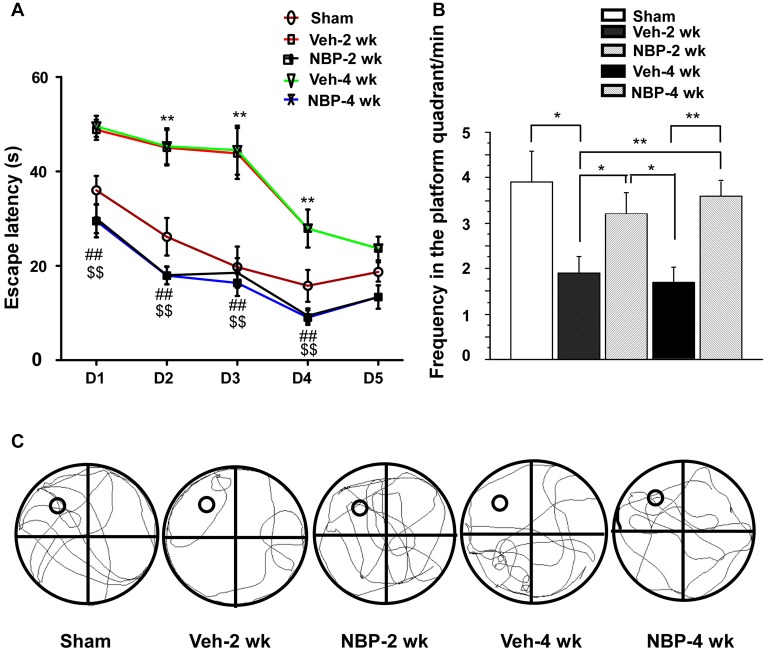
Alteration of cognitive impairment as measured by the Morris water maze (MWM) in response to vehicle and NBP treatment. **(A)** Escape latency changes in the water maze in the different groups from day 1 to day 5. (**p < 0.01, Veh-2 week group or Veh-4 week group vs. the sham group; ^##^*p* < 0.01, NBP-2 week group vs. Veh-2 week or Veh-4 week groups; ^$$^*p* < 0.01, NBP-4 week group vs. Veh-2 week or Veh-4 week groups). **(B)** Changes in the frequency of time in the platform quadrant between the different groups. **p < 0.01; *p < 0.05. **(C)** Swimming path of rats at day 6 in the different groups. The small empty circle represents the platform in one quadrant of the swimming pool (the large circle). The swimming path distance in the platform quadrant was reduced in the Veh-2 week or Veh-4 week groups compared to the sham, NBP- 2 week or NBP- 4 week groups.

Finally, we investigated whether NBP could improve cognitive impairment induced by BCCAO using the MWM. For this assessment, we analyzed the escape latency and the frequency in the platform quadrant. Plots of escape latency in each group are shown in Figure 7A, which illustrates a gradual decreased latency pattern in the final days of training. However, the escape latency was notably reduced in NBP-treated rats at 2 weeks and 4 weeks after BCCAO from day 1 to day 4 when compared to the vehicle-treated group (all *p* < 0.01). In addition, the escape latency for vehicle-treated rats was significantly longer than that for the sham group from day 2 to day 4 in the 2 weeks and 4 weeks groups (all *p* < 0.01). The frequency of time in the platform quadrant at day 6 was 3.90/min ± 0.67 in the sham group but significantly less in the vehicle-treated group at 2 weeks (1.90/min ± 0.38, *p* < 0.05) and 4 weeks (1.70/min ± 0.34, *p* < 0.01) after BCCAO when compared to the sham group, respectively (Figure 7B). However, after treatment with NBP, the frequency increased to 3.2/min ± 0.47 at 2 weeks and 3.60/min ± 0.31 at 4 weeks after BCCAO (*p* < 0.05 and *p* < 0.01, respectively, vs. vehicle-treated group) after BCCAO. At day 6, the swimming path of rats was different between the different groups (Figure 7C). The swimming path in the platform quadrant was less in the Veh-2 week or Veh-4 week groups than that in the sham, NBP- 2 week or NBP- 4 week groups.

## Discussion

The present study provides the first report of NBP’s effects on MRI-documented CBF dynamic changes in an experimental CCH model. In addition, NBP treatment also protects neurons against neuronal degeneration, reduces apoptosis and glial reactivity and cognitive impairment in CCH caused by BCCAO.

### Effects of NBP on Dynamic Changes in CBF and Vascular Plasticity in CCH Rats

Our previous research has shown that cortical and striatal CBF dramatically decreased acutely and returned to the sham level from 3 weeks after BCCAO (Jing et al., 2015). In addition, we also found that BCCAO triggered gradual dilation of VAs and increased microvascular density in the cortex, striatum and cerebellum at 4–6 weeks after BCCAO. Although this phenomenon is part of a compensatory response to insufficient CBF in the forebrain, this compensatory ability is limited and fails to prevent neurodegeneration and amelioration of memory impairment (Jing et al., 2015). In the present study, however, we found that NBP was able to significantly elevate CBF level in the cortex, striatum and hippocampus from an early stage (1 week) and returned it to a normal level at 2 weeks following BCCAO. Meanwhile, in the vehicle group, it took 4 weeks post-BCCAO for CBF to reach the pre-occlusion level. The mechanism underlying recovery of CBF by NBP treatment may be related to a subsequent increase in VA diameter and promotion of angiogenesis following BCCAO (Figures 3, 4). Also, NBP treatment prevented neuronal loss and improved memory deficits, which may be related to the earlier recovery of CBF by NBP (Figure 2). It has been reported that NBP can prevent cold-induced ischemic stroke via improvement of cerebral microvasculature (Liu et al., 2007). The underlying mechanism by which NBP promotes angiogenesis under CCH conditions is not completely clear. Studies have shown that NBP can up-regulate expression of VEGF in diabetic rats (Zhang et al., 2010) and Alzheimer’s rats (Hou et al., 2010) and expression of VEFG and BFGF in focal cerebral ischemic rats (Li et al., 2008). Therefore, it is plausible that the effect of NBP on angiogenesis in CCH rats may be regulated by increasing expression of VEGF and BFGF. In addition, NBP can also promote microcirculation by inhibiting activation of platelet-forming pathways (Ye et al., 2015). Further investigation is required to clarify this question.

### Effects of NBP on Neuroprotection, Glial Reactivity and Memory Amelioration in CCH Rats

Many studies have shown that CCH causes neurodegeneration, glial relativity and memory deficits in experimental models as well as in patients with subcortical ischemic vascular dementia (SIVD) and AD [32, 2]. AD and SIVD patients showed marked CBF reductions in the forebrain (Schuff et al., 2009). Using CCH rats induced by BCCAO, we have previously found that neuronal degeneration, inflammation and cognitive impairment are prominent in animals from 2 weeks to 6 weeks following BCCAO, and the extent of cognitive dysfunction is dependent on the duration of ischemia (Jing et al., 2015). This further highlights the critical need for effective therapies for patients suffering from chronic hypoxic/ischemic stroke.

NBP has been proven to play a critical role in neuroprotection, anti-inflammation, anti-apoptosis and amelioration of memory deficits in ischemic animal models and stroke patients. It has been reported that NBP protects immortalized human umbilical vein endothelial cells (HUVECs) and rat brain microvascular endothelial cells from death by blocking oxidative/nitrosative stress and mitochondrial damage when these cells are exposed to oxygen glucose deprivation (OGD) *in vitro* (Li et al., 2009a; Yang et al., 2012). NBP also reduces neuronal cell death, significantly lowers neurological deficit scores and reduces infarct volume in the penumbra of MCAO rats (Li et al., 2010; Zhang et al., 2012). In a transient cardiac arrest rat model, NBP shows protective benefits for the majority of CA1 neurons against death (Zhang et al., 2016). Based on multiple reports of effective outcomes in animal studies, NBP has been proven to be effective in treating stroke patients in several clinical trials in China. One study showed that patients (20) suffering from AIS exhibit lower NIHSS scores than the placebo group (20 AIS patients) but the NIHSS scores are similar to those in Cerebrolysin group (20 AIS patients) after receiving NBP treatment within 12 h after AIS for 10 days (Xue et al., 2016). This study also demonstrates the safety of usage of NBP. Another large scale clinical study covered 38 hospitals from 2007 to 2009, during which 573 patients suffering from AIS within 48 h of stroke onset were enrolled to receive a 90 day treatment with NBP alone, NBP plus aspirin, or ozagrel plus aspirin (Cui et al., 2013). The best outcome reported from this study was achieved from the group treated only with NBP. Furthermore, patients with AIS have higher level of circulating progenitor cells after NBP treatment (Zhao et al., 2016). Therefore, from the results of AIS in experimental animals and patients, NBP treatment has achieved satisfying outcomes in reducing neuronal death, ameliorating neurological deficits and stimulation of microcirculation. The mechanisms of neuroprotection mediated by NBP in acute ischemic injury may occur via multiple pathways including anti-oxidation, anti-apoptosis and anti-inflammation (Li et al., 2009a, 2010; Yang et al., 2012).

The outcomes of the present study further demonstrate that NBP treatment can also protect hippocampal neurons, reduce apoptosis and astrocyte reactivity and improve spatial memory deficits in CCH rats. These results suggest that NBP may be also a promising drug for CCH or vascular dementia. It would be interesting to explore the additional effects of NBP on other diseases such as vascular dementia or AD.

### Neurodegeneration in Acute and Chronic Global Ischemia Models

Transient global ischemia can be achieved by occlusion of bilateral common carotid arteries and VAs (4-VO ischemia model; Pulsinelli et al., 1982; Yamaguchi et al., 2005). It has been well established that transient global ischemia model induces selective neuronal death (Pulsinelli et al., 1982; Traystman, 2003; Yamaguchi et al., 2005). The highest mortality of neurons is found in the CA1 of the hippocampus followed by cortical and striatal spiny neurons after transient global ischemia (Pulsinelli et al., 1982). In Pulsinelli et al.’s (1982) study, 10 min ischemia causes approximately 50% CA1 neurons with severe damage, whereas approximately 50% neurons with moderate damage are found in the CA3 area after 20 min ischemia. Therefore, CA1 pyramidal neurons are ischemia-sensitive, whereas CA3 pyramidal neurons are relatively resistant in the acute transient global ischemia. In contrast, permanent occlusion of bilateral common carotid arteries (2-VO) causes chronic hypoperfusion (Farkas et al., 2007). Neurodegeneration in 2-VO model is not as severe as that in the acute 4-VO model which may be due to hemodynamic compensatory responses of the brain. It has been reported that the hippocampal size did not change in CCH brain, but the neuronal density as determined by NeuN staining in the CA1 area is significantly reduced at 7 days after BCCAO (Cechetti et al., 2012). Surprisingly, in the present study, we found that the number of NeuN positive neurons is reduced not only in the CA1 area, but also in the CA3 areas at 2 weeks and 4 weeks after BCCAO, suggesting that the neurodegenerative mechanisms induced by acute transient global ischemia and CCH are distinct, and further studies are obviously important in order to appropriately use these ischemic models and to identify specific and effective therapeutic agents for treating different types of ischemia.

## Author Contributions

LH, YR designed and organized the project, revised and approved the manuscript. ZX, WL and LHZ performed experiment, collected and analyzed data and wrote the manuscript. CKT revised and approved the manuscript. LZ, CS, ZJ, YX and WL performed experiment, collected and analyzed data.

## Conflict of Interest Statement

The authors declare that the research was conducted in the absence of any commercial or financial relationships that could be construed as a potential conflict of interest.
